# Potential of Cricket Chitosan for Nanoparticle Development Through Ionotropic Gelation: Novel Source for Cosmeceutical Delivery Systems

**DOI:** 10.3390/pharmaceutics16121618

**Published:** 2024-12-20

**Authors:** Jirasit Inthorn, Pratthana Chomchalao, Puracheth Rithchumpon, Saranya Juntrapirom, Watchara Kanjanakawinkul, Thomas Rades, Wantida Chaiyana

**Affiliations:** 1 Department of Pharmaceutical Sciences, Faculty of Pharmacy, Chiang Mai University, Chiang Mai 50200, Thailand; jirasit_i@cmu.ac.th; 2 College of Medicine and Public Health, Ubon Ratchathani University, Ubon Ratchathani 34190, Thailand; pratthana.c@ubu.ac.th; 3 Department of Chemistry, Faculty of Science, Khon Kaen University, Khon Kaen 40002, Thailand; purarit@kku.ac.th; 4 Chulabhorn Royal Pharmaceutical Manufacturing Facilities by Chulabhorn Royal Academy, Chon Buri 20180, Thailand; saranya.jun@cra.ac.th (S.J.); watchara.kan@cra.ac.th (W.K.); 5 Department of Pharmacy, Faculty of Health and Medical Sciences, University of Copenhagen, Universitetsparken 2, 2100 Copenhagen, Denmark; thomas.rades@sund.ku.dk; 6 Center of Excellence in Pharmaceutical Nanotechnology, Faculty of Pharmacy, Chiang Mai University, Chiang Mai 50200, Thailand; 7 Multidisciplinary and Interdisciplinary School, Chiang Mai University, Chiang Mai 50200, Thailand; 8 Research Center of Deep Technology in Beekeeping and Bee Products for Sustainable Development Goals (SMART BEE SDGs), Chiang Mai University, Chiang Mai 50200, Thailand

**Keywords:** *Gryllus bimaculatus*, *Teleogryllus mitratus*, *Acheta domesticus*, cricket, chitosan, nanoparticle, ionotropic gelation

## Abstract

Background/Objectives: Crickets are recognized as an alternative source of chitosan. This study aimed to assess the potential of cricket-derived chitosan as a natural source to develop chitosan nanoparticles (CNPs). Methods: Chitosan were isolated from different cricket species, including *Gryllus bimaculatus*, *Teleogryllus mitratus*, and *Acheta domesticus*. The isolated chitosan were characterized by their functional groups, crystallographic and thermal properties, molecular structure, morphology, water solubility, molecular weight, binding capacity, irritation potential, and cytotoxicity in comparison to commercial shrimp-based chitosan. CNPs were developed through an ionotropic gelation method, followed by the evaluation of particle size, polydispersity index (PDI), and zeta potential. Results: The findings of this study indicate that chitosan can be successfully isolated from the three cricket species, with yields ranging from 4.35% to 5.22% *w*/*w* of the dried material. The characteristics of cricket-based chitosan were similar to those of commercial chitosan, except that the cricket-based chitosan displayed a higher crystallinity and a lower molecular weight. Additionally, CPNs were successfully produced from cricket-based chitosan using sodium citrate as a crosslinking agent. All cricket-based chitosan exhibited no irritation or cytotoxicity. Chitosan derived from *A. domesticus* however was found to be the most suitable to develop CPNs, as it produced the smallest particle size (522.0 ± 12.1 nm) with a comparatively narrow PDI (0.388 ± 0.026) and an acceptable positive zeta potential (34.2 ± 4.4 mV). Conclusions: Cricket-derived chitosan compares favorably with crustacean-derived chitosan and showed potential for a range of applications, including the use as a nanocosmeceutical delivery system in topical and cosmetic formulations.

## 1. Introduction

Crickets, edible insects, are descendants of the Gryllidae family, which belongs within the class of Insecta and the Orthoptera order. Thailand currently promotes cricket farming for nutrition as well as an alternative food (protein) source, focusing on the three species: the field or two-spotted cricket (*Gryllus bimaculatus*), the ground cricket (*Teleogryllus mitratus*), and the house cricket (*Acheta domesticus*) [1,2]. Despite their lack of recognition in the past, crickets are a potential and enduring source of chitin and chitosan. In this context, crickets possess some advantages over crustaceans. They are not influenced by seasonal changes and can be efficiently bred in large quantities because of their short life span and rapid rate of reproduction [3]. The food industry uses several techniques to separate fat and chitin from protein during the cricket protein extraction process [1]. The majority of cricket protein extraction protocols rely on aqueous fractionation [4]. Therefore, chitin-rich solid fractions are by-products of protein extraction, which offer an appealing opportunity to add value to these by-products.

Chitin, a common and abundant natural polysaccharide found in the exoskeletons of shrimps, crabs, fungus cell walls, and insects, can be converted into chitosan through deacetylation using either alkaline hydrolysis or enzymatic methods [5]. Chitosan is a copolymer consisting of two structural components, including N-acetyl-D-glucosamine and D-glucosamine. Chitosan properties differ depending on their degree of deacetylation, e.g., their solubility properties in acidic conditions [6]. Chitosan is a promising and versatile ingredient as a moisturizer, thickener, and film-forming agent in the cosmetic industry that shows potential to enhance the condition and appearance of skin. Additionally, chitosan is an encapsulating agent in cosmeceuticals due to its numerous benefits, including its low toxicity, high compatibility with skin, and biodegradability [7].

The cosmeceutical industry increasingly employs nanocosmeceuticals to address skin problems, accounting for a significant market share [8]. In the area of cosmetics and cosmeceuticals, nano delivery systems, also known as nanocarriers, are an innovative area of research that involves the development, analysis, production, and application of materials and systems at the nanoscale level (1–1000 nm) for a variety of purposes [9]. Nanocosmeceuticals are also a representation of beneficial features of topical products, such as increased efficacy, improved physical and chemical stability, active transport of active ingredients, occlusive properties, and controlled release [10]. Chitosan nanoparticles (CNPs) are commonly employed in the field of drug delivery systems for dermal application due to their simple fabrication process and scalable preparation characteristics [11]. Ionotropic gelation methods are the most frequently used for CNP production techniques. Typically, negatively charged chemicals are used as a crosslinking agent to form complex coacervates with positively charged chitosan in an acidic aqueous phase [12].

Chitosan derived from insects is gaining popularity as an alternative to that derived from crustaceans. Nevertheless, there is an insufficient amount of information regarding chitosan derived from crickets. Despite the promotion of three cricket species for farming, chitosan in the by-products derived from cricket protein extraction remains unexploited in nanocosmeceutical delivery systems [1]. This investigation used a chemical fabrication procedure to extract chitosan from the defatted cricket powder, which then served as the core material for CNP development. Although a few studies were conducted to extract and evaluate cricket chitosan, there is no study to compare the chitosan of the three cricket species in terms of its use as a nanocarrier for cosmeceutical ingredients [10]. Therefore, the primary objective of the present research is to extract and characterize the chitosan obtained from three cricket species, with a particular emphasis on investigating their physicochemical properties, and safety assessment focusing on examining irritation and cytotoxicity. CNPs were developed using the ionotropic gelation process with cricket chitosan as the core material. Chitosan from cricket was then characterized and applied as a novel source for nanocosmeceutical delivery systems.

## 2. Materials and Methods

### 2.1. Materials

#### 2.1.1. Crickets

Three species of crickets, i.e., *Gryllus bimaculatus* (GB), *Teleogryllus mitratus* (TM), and *Acheta domesticus* (AD), after reaching their adulthood (approximately 45 days old), were purchased in frozen form from the Yimsoo Farm at Mae Rim, Chiang Mai, Thailand. The frozen crickets were subjected to lyophilization in a freeze dryer (Beta 2–8 LD-plus, Christ, Osterode am Harz, Germany) at −50 °C for 24 h.

#### 2.1.2. Chemicals

Commercial shrimp chitosan (CSC) with a specification of 75–85% degree of deacetylation, low molecular weight (CAS Number 9012 76-4), and polysorbate 80 (Tween^®^ 80) were supplied by Sigma-Aldrich (Munich, Germany). Absolute ethanol, citric acid, dimethyl sulfoxide (DMSO), glacial acetic acid, hydrochloric acid (HCl), sodium citrate, sodium hydroxide (NaOH), and sodium tripolyphosphate, all of analytical grade, were provided by RCI Labscan Ltd. (Bangkok, Thailand). Gum arabic was purchased from KemAus (Cloisters Cherrybrook, Australia). Mineral oil, soybean oil, sodium chloride, and sodium lauryl sulfate were cosmetic grade and purchased from Namsiang Co., Ltd. (Bangkok, Thailand). 3-(4,5-Dimethylthiazol-2-yl)-2,5-diphenyl-2H-tetrazolium bromide (MTT) was purchased from Tokyo chemical industry Co., Ltd. (Tokyo, Japan). Crystal violet was purchased from Riedel-de Haen (Munich, Germany). Fetal bovine serum, penicillin, streptomycin, and Dulbecco’s modified eagle medium/nutrient mixture F-12 (DMEM/F12) medium were purchased from GIBCO Life Sciences Corp. (Grand Island, NY, USA).

### 2.2. Experimental Methods

#### 2.2.1. Defatted Cricket Powder and Chitosan Preparation

The dried cricket materials were subjected to cold pressing through an oil screw press extraction machine (FEA-100SS-M-H-TC, Energy Friend Ltd., Part., Chiang Mai, Thailand) to eliminate the oil and fat, as described earlier [13]. The cricket residues from the oil extraction process were pulverized into a powder by a commercial blender (Powder Grinder PG500, Spring Green Evolution Co., Ltd., Bangkok, Thailand). Subsequently, the cricket powder was defatted using absolute ethanol. After dispersing the cricket powder in absolute ethanol at a solvent-to-powder ratio of 1:20 and stirring for 4 h with the solvent replaced at 2 h intervals [14], the defatting solvent was removed after filtration through a Whatman^®^ filter paper, Grade 4. The defatted powders were then collected and left to dry at room temperature [15]. The defatted powders were collected in a closed container for further experiments.

Cricket chitosan was extracted using a chemical modification method from He et al. [5] and Ibitoye et al. [16]. For deproteinization, 50 g of the defatted powders were treated with 500 mL of a 1.0 M NaOH solution at 80 °C for 6 h under moderate stirring on a hot plate stirrer with temperature controlled by an electronic contact thermometer (IKA^®^ C-MAG HS7, IKA Werke GmbH & co. KG, Staufen, Germany). Deproteinized chitin pellets obtained through filtration were further washed with distilled water until the washing water reached a neutral pH. For demineralization, deproteinized chitin pellets were subjected to 500 mL of a 0.25 M HCl solution for 15 min at the maintained temperature of 85 °C. Subsequently, the resulting demineralized chitin pellets were further washed until a neutral pH was reached and left to dry. For deacetylation, the acetyl groups of the demineralized chitin pellets were removed by soaking in a NaOH solution (67% *w*/*v*) at a powder-to-solvent ratio of 1:20. The temperature of the mixture was maintained at 110 °C for 2 h with moderate stirring. The deacetylated chitosan pellets (chitosan derived from cricket) were filtrated and washed until a neutral pH was achieved. After that, the cricket chitosan was dried overnight at 60 °C. The percentage yield was calculated using the following equation:

Yield (%) = (*A*/*B*) × 100 (1)

where *A* is the weight of the cricket chitosan and *B* is the weight of the cricket defatted powders. All experiments were performed in independent triplicate.

#### 2.2.2. Characterizations of Cricket Chitosan

The cricket chitosan from the three cricket species was characterized in terms of their functional bonds, thermal characteristics, and morphology, with CSC as a reference substance.

- Fourier-transform infrared (FT-IR) spectroscopy

The presence of different infrared bands in cricket chitosan was determined in transmittance mode using FT-IR spectroscopy (ALPHA II, Bruker, Karlsruhe, Germany) to identify its functional bonds. The FT-IR spectra were scanned across the wavenumber range of 4000 to 400 cm^−1^ and graphed with transmittance on the Y-axis and wavenumber (cm^−1^) on the X-axis.

- X-ray diffraction (XRD)

The XRD pattern of the cricket chitosan was measured using an X-ray diffractometer (D2 PHASER, Bruker, Karlsruhe, Germany) operated at 30 kV and 10 mA. The chitosan powders were placed on the XRD sample holder and scanning was initiated at 2-theta angles from 5 to 80°, with a step duration of 0.2°·s^−1^. The XRD diffractograms were graphed utilizing intensity values (in arbitrary units) on the Y-axis and the 2-theta angle (in degrees) on the X-axis [16].

- Thermogravimetric analysis (TGA)

The thermal characteristics of the cricket chitosan were determined by analyzing the weight loss profile using a TA Q 50 system (TGA550, TA Instruments, New Castle, DE, USA). Cricket chitosan of approximately 10 mg was heated from 50 to 800 °C at a heating rate of 10 °C/min under an inert atmosphere of nitrogen with a flow rate of 60 mL/min. The thermograms were graphed with the Y-axis representing the percentage of weight and the X-axis representing the temperature.

- Differential scanning calorimetry (DSC)

The DSC thermogram of cricket chitosan was determined by using a DSC Q1000 V7.0 A universal (DSC Q1000, TA Instruments, New Castle, DE, USA) under a nitrogen atmosphere. Around 5–8 mg of cricket chitosan was sealed in aluminum pans and heated at 10 °C/min from 50 °C to 500 °C. The thermograms were graphed with the Y-axis representing the heat flow and the X-axis representing the temperature [17].

- Scanning electron microscopy (SEM)

The morphology of the cricket chitosan was observed at magnifications of 500 and 5000 using SEM (TESCAN CLARA, TESCAN, Brno-Kohoutovice, Czech Republic). The cricket chitosan pellets were mounted on a metal stub using a sticky carbon disk, increasing conductivity. Before scanning, the cricket chitosan pellets were coated with a thin layer of gold (Au) at 8.0 nm of thickness using a sputter coater (SAFEMATIC model CCU-010, Zizers, Switzerland). Morphological analysis of the cricket chitosan was performed to investigate the surface, size, shape, and form of the pellets [14].

- Proton nuclear magnetic resonance (^1^H-NMR) spectroscopy

The proton species present in the cricket-derived chitosan were characterized using ^1^H-NMR spectroscopy. NMR spectra were acquired on a Bruker AM400 spectrometer operating at 400 MHz. The samples were dissolved in the specified solvents, and the solvent residual peak (H_2_O impurity in D_2_O, δ 4.80 ppm) served as an internal reference. The ^1^H-NMR spectra were plotted with chemical shift (δ, ppm) on the X-axis and intensity on the Y-axis.

#### 2.2.3. Solubility Assessment

The solubility of the cricket chitosan was assessed in several solvents, including deionized water, absolute ethanol, DMSO, mineral oil, and buffer solutions with pH values of 1.0, 3.0, 5.0, 7.0, and 9.0, as described in a previous study [13]. Briefly, an exact quantity of cricket chitosan was accurately weighed, and the solvents were dropwise added until the cricket chitosan was completely dissolved with the assistance of a vortex mixer (Genie 2, Scientific Industries Inc., New York, NY, USA). The solubility of each cricket chitosan was determined by applying the following equation:

Solubility (mg/mL) = *A*/*B* (2)

where *A* is the weight of the cricket chitosan in milligrams (mg) and *B* is the amount of the solvent needed to completely dissolve the chitosan in milliliters (mL). The experiments were conducted independently, with each experiment replicated three times.

#### 2.2.4. Determination of Water and Fat Binding Capacities

The water and fat binding capacities of the cricket chitosan were measured using a method described by Rashid et al. [18] with modifications. Firstly, the water binding capacity was determined by mixing 0.5 g of cricket chitosan with 10.0 mL of distilled water, followed by thorough mixing using a vortex mixer (Genie 2, Scientific Industries Inc., New York, NY, USA) for 1 min. The resulting mixture was mixed using a shaking mixer with a speed set at 30 rpm for 30 min at an ambient temperature, followed by centrifugation at 3500× *g* for 25 min. Subsequently, the supernatant was decanted, followed by the weighing of the water-bound cricket chitosan. The fat binding capacity of cricket chitosan was determined using the same method but with soybean oil used instead of water. The water and fat binding capacities were calculated as follows:

Water binding capacity (%) = (*A*/*B*) × 100 (3)

Fat binding capacity (%) = (*C*/*B*) × 100 (4)

where *A* is the weight of the water-bound cricket chitosan after decanting the water supernatant, *B* is the initial weight of cricket chitosan, and *C* is the weight of the water-bound cricket chitosan after decanting the soybean oil supernatant.

#### 2.2.5. Determination of Molecular Weight

The molecular weight of cricket chitosan was determined using the viscometric method, as previously described by Malm et al. [19]. Firstly, 0.2–1.0% *w*/*v* of cricket chitosan was dissolved in a mixture of 0.1 M acetic acid solution and 0.2 M sodium chloride solution in a volume ratio of 1:1. The resulting solutions were injected into an Ostwald viscometer that was placed in a water bath set at a constant temperature of 25 °C. The duration that cricket chitosan solutions passed through a capillary was recorded. Specific viscosity (η_sp_) and reduced viscosity (η_red_) were calculated using the recorded duration [20]. The intrinsic viscosity (η) was determined by plotting a graph of the reduced viscosity (η_red_) against concentration to a concentration of zero [20]. The Mark–Houwink equation, as shown in the following equation, was used to calculate the molecular weight of cricket chitosan.

Intrinsic viscosity (η) = *K*(*Mv*)*^a^* (5)

where *K* is 1.81 × 10^−3^, *Mv* is the molecular weight, and *a* is 0.93, which are empirical constants that depend on the polymer nature, the solvent, and the temperature [18].

#### 2.2.6. Determination of Irritation Potency and Cytotoxicity of Cricket Chitosan

- Hen’s Egg Chorio-Allantoic Membrane (HET-CAM) test

The irritant potential of cricket chitosan was evaluated through an HET-CAM test using fertilized hen’s eggs that were incubated in a constant temperature of 38.3 ± 0.2 °C and a relative humidity of 58 ± 2% with a five-times-per-day rotation until day nine [13]. The eggshell around the area marked as the air cell was opened using a rotating dental saw blade and carefully removed with care to avoid injuring the inner membrane. Subsequently, a normal saline solution was applied to the inner membrane to moisten it before removal using forceps. After that, 30 μL of a 10 mg/mL cricket chitosan solution was applied directly onto the CAM. The reactions on the CAM were observed over a period of 300 s. Irritation signs, in terms of hemorrhage (bleeding from the vessels), vascular lysis (blood vessel disintegration), and coagulation (intra- and extra-vascular protein denaturation), were observed under the stereoscope (NS80, Ningbo Yongxin Optics, Ningbo, China) for 300 s. An irritation score (IS) was calculated using the following equation:

Irritation score = [(301 − *h*) × 5]/300 + [(301 − *l*) × 7]/300 + [(301 − *c*) × 9]/300 (6)

where *h*, *l*, and *c* represent the durations in terms of seconds for the initial bleeding, vascular breakdown, and blood clotting, respectively. The irritation score (*IS*) can be classified into four distinct categories: non-irritation (0.0–0.9), mild irritation (1.0–4.9), moderate irritation (5.0–8.9), and severe irritation (9.0–21.0) [13]. A sodium lauryl sulfate aqueous solution at a concentration of 1% *w*/*v* and normal saline solution were used as positive and negative controls, respectively. The experiments were implemented in duplicate.

- 3-(4,5-Dimethylthiazol-2-yl)-2,5-diphenyl-2H-tetrazolium bromide (MTT) assay

The effects of cricket chitosan on human dermal fibroblast (NHDF) cytotoxicity and cell proliferation were determined by the MTT assay modified from Muniandy et al. [21]. In brief, the NHDF cells were cultured in DMEM-F12 supplemented with 10% fetal bovine serum and 1% penicillin/streptomycin were trypsinized and seeded at a density of 1 × 10^4^ cells per well in a 96-well plate and incubated at a temperature of 37 °C and a 5% CO_2_ for 24 h. Subsequently, the culture medium was discarded and washed with sterile phosphate buffer pH 7.4. The cells were exposed to the medium with and without various concentrations of cricket chitosan solution. After 24 h of treatment, the medium was carefully removed and the NHDF cells were washed with sterile phosphate buffer 7.4. Subsequently, NHDF cells were exposed to an MTT solution and placed in the dark at 37 °C for 2 h. The medium was removed, and the formazan crystals formed by metabolically viable cells were dissolved using DMSO. The absorbance for each test sample was measured using a microplate reader (CLARIOstar^®^, BMG LABTECH, Ortenberg, Germany) at a wavelength of 570 nm. The percentage of cell viability was calculated using the following equation:

Cell viability (%) = (*A*/*B*) × 100 (7)

where *A* is the absorbance of the reaction with cricket chitosan at 570 nm and *B* is the absorbance of the control without the cricket chitosan.

- Crystal violet staining

The morphology and density of the NHDF cells were evaluated using a crystal violet staining assay [22]. In brief, the NHDF cells were cultured and exposed to various concentrations of the cricket chitosan solution, similar to the MTT assay. After 24 h, the media were removed and the NHDF cells were washed with phosphate buffer pH 7.4, followed by fixing with 4% paraformaldehyde for 1 h at room temperature. After fixation, the NHDF cells were treated with a 0.5% crystal violet solution and incubated for 30 min. Subsequently, the NHDF cells were washed with tap water to eliminate excess stains and dried in a normal atmosphere. The cell morphology and density were observed and captured by light microscope (EVOS^®^ XL Core Imaging System, Thermo Fisher Scientific Inc., Waltham, MA, USA).

#### 2.2.7. Development of CNPs

- Optimization of CNP formulation

1. 1. Effect of crosslinking agent and CSC concentration

The CNPs were prepared on the basis of the ionotropic gelation method, with some modifications [23,24]. CSC and polysorbate-80 were dissolved in 75 mL of a 0.2% *v*/*v* acetic acid solution and then homogenized using a high shear homogenizer (T25 ULTRA-TURRAX^®^ digital, IKA Werke GmbH & co. KG, Staufen, Germany) at 3000 rpm for 5 min. Citric acid, gum arabic, sodium citrate, and sodium tripolyphosphate were used as crosslinking agents. The crosslinking agent was dissolved in 25 mL of distilled water and then dropped into the CSC solution with a 1 mL/min flow rate under continuous homogenization at 3000 rpm.

2. Effect of cricket chitosan concentrations

The CNP formulation from cricket chitosan was optimized using the same procedure as described above, except cricket chitosan was used instead of CSC and sodium citrate was used as a crosslinking agent. The CSC was also used as a reference chitosan in this experiment.

- Characterization of CNPs

The physical appearance of each CNP dispersion was evaluated by visual inspection. Furthermore, the mean particle size and polydispersity index (PDI) were investigated using dynamic light scattering, while the zeta potentials were analyzed using the electrophoretic light scattering technique (Zetasizer version 5.00, Malvern Instruments Ltd., Malvern, UK) with a disposable folded capillary cell (DTS1070, Malvern Instruments Ltd., Malvern, UK). The results are shown as average and standard deviation (SD) calculated from independent triplicate samples.

- Stability test for CNPs from cricket chitosan

CNP formulations were evaluated for their physical stability by storing them in the dark at room temperature for 60 days. Subsequently, the CNPs were characterized for their physical appearance and sedimentation. Particle size, PDI, and zeta potentials were also measured at the start (day 0) and at the end (day 60) of the study.

### 2.3. Statistical Analyses

The data are presented as the mean value and standard deviation. The statistical significance of the data was assessed using GraphPad Prism version 10.2.3 by the application of one-way analysis of variance (ANOVA) and Tukey’s post hoc tests (GraphPad Software Inc., La Jolla, CA, USA), whereas a paired sample t-test was conducted using GraphPad Prism to compare the two sets of samples. A *p*-value less than 0.05 signifies statistical significance.

## 3. Results and Discussions

### 3.1. Cricket Materials and Cricket Chitosan

**Figure 1:**
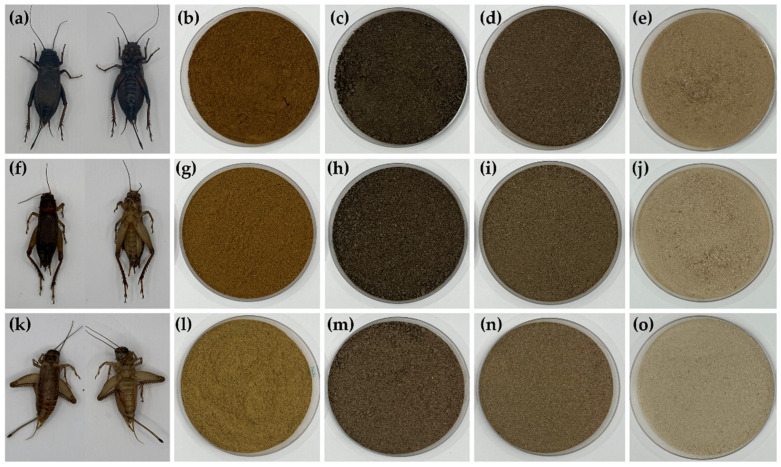
Dorsal and ventral views of *G. bimaculatus*, GB (**a**); *T. mitratus*, TM (**f**); and *A. domesticus*, AD (**k**); along with their corresponding defatted powders (**b**,**g**,**l**); deproteinized chitins (**c**,**h**,**m**); demineralized chitins (**d**,**i**,**n**); and deacetylated chitosan (**e**,**j**,**o**).

In the current investigation, three cricket species were used: GB, TM, and AD. The observed variations across cricket species specifically illustrate differences in size and body color, as shown in Figure 1. GB had the largest body size, followed by TM, while AD was the smallest. GB was an ebony black, TM was blackish-brown, and AD was yellowish-brown. The gradient shades of colors of crickets’ bodies are caused by the distribution of melanin and sclerotin pigments in the cuticles [25]. Defatted powder was obtained after pulverizing the solid fraction from the oil extraction process, as described in a previous study [13]. Cricket chitosan from the three cricket species was successfully extracted. Defatted powder, deproteinized chitin, demineralized chitin, and deacetylated chitosan were small pellets displaying shading colors corresponding to the cuticle color of each cricket species, as shown in Figure 1. Chitosan from AD showed the lightest color when compared to those from GB and TM. The presence of melanin in cricket chitosan is responsible for its tan color, which is why it appears darker and more yellow compared to commercial shrimp chitosan [26]. The result demonstrates that the color of deacetylated chitosan from AD is the most comparable to that of commercial chitosan from shrimp.

**Figure 2:**
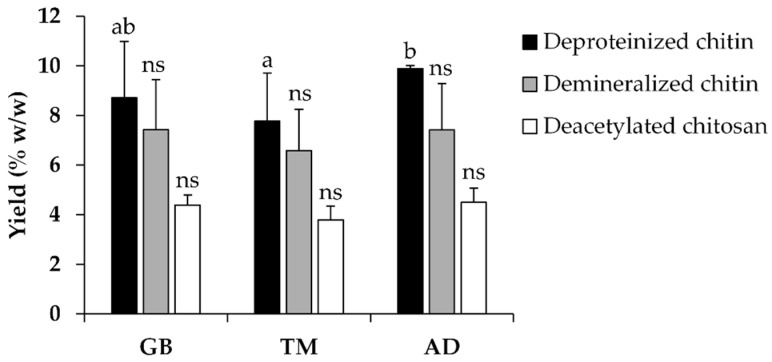
Yields of deproteinized chitin (■), demineralized chitin (■), and deacetylated chitosan (☐) from *G. bimaculatus* (GB), *T. mitratus* (TM), and *A. domesticus* (AD). Differing letters (a and b) indicate significant differences among cricket species, as determined by one-way ANOVA followed by Tukey’s multiple comparison test (*p* < 0.05), while ns indicates no significant difference.

The deproteinized chitin, demineralized chitin, and deacetylated chitosan yields are shown in Figure 2 and Table 1. Deacetylated chitosan yield from GB, TM, and AD were 5.22 ± 0.99, 4.35 ± 0.72, and 5.17 ± 0.89% on a dry basis, respectively. Similarly, a previous study reported that the yield of chitin from AD was 5.7% [26], ranging from 4.3% to 7.1% [16]. From these chitosan extraction results, it can be concluded that deproteinization with NaOH is very efficient as crickets contain approximately 63.3–71.09% protein on dry base (including fat) [27,28]. Moreover, demineralization with HCl can effectively remove inorganic material from defatted powder, approximately 1.12–2.41% of dry bases, because insects have lower levels (less than 10%) of inorganic material compared to crustaceans [29].

### 3.2. Characterization of Cricket Chitosan

**Figure 3:**
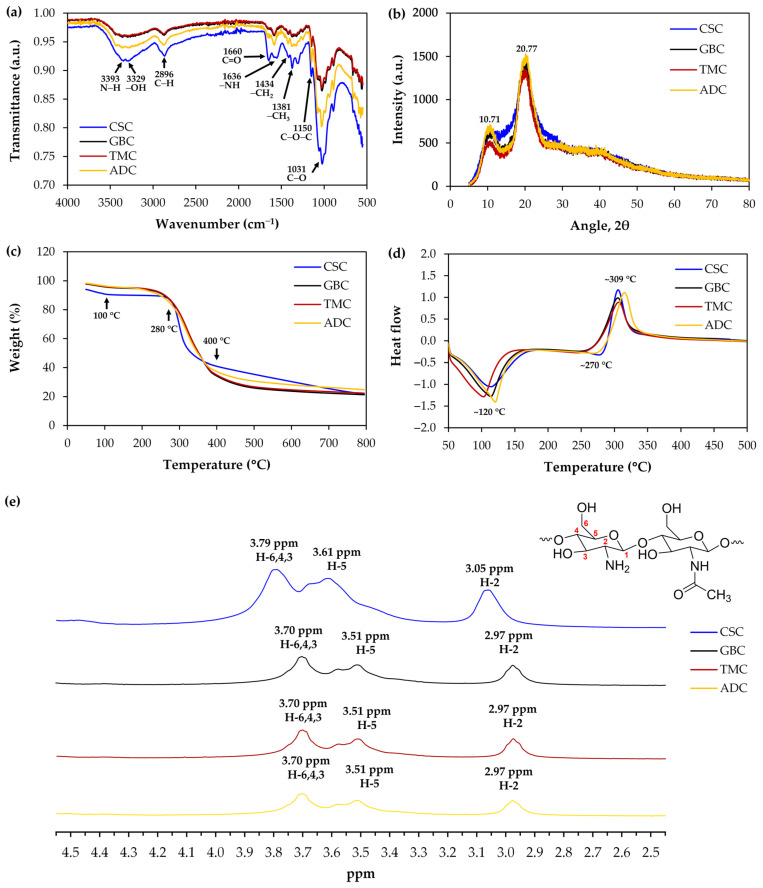
FT-IR spectra (**a**), XRD diffractograms (**b**), TGA thermograms (**c**), DSC thermograms (**d**), and ^1^H-NMR spectra (**e**) of commercial shrimp chitosan (CSC), compared to chitosan extracted from *G. bimaculatus* (GBC), *T. mitratus* (TMC), and *A. domesticus* (ADC). The chemical structure of chitosan, with carbon atoms labeled with red numbers (1–6) corresponding to C1, C2, C3, C4, C5, and C6, respectively.

FT-IR analysis of all cricket chitosan revealed strong chemical and structural parallels to CSC, as shown in Figure 3a. The cricket chitosan in this investigation exhibited FT-IR spectra that were comparable to those published by others [16,17,19], indicating that the 3393, 3329, 1660, 1603, and 1381 cm^−1^ characteristic bands of chitin and chitosan can be attributed to N–H stretching, O-H stretching, C=O stretching, N−H stretching in NHCOCH3 group (amide II band), and -CH3 stretching, respectively.

The XRD diffractograms of cricket chitosan compared to CSC are shown in Figure 3b. The diffractogram illustrates the existence of two prominent peaks at 10.71°2*θ* and 20.77° in 2*θ*, as reported by Jampafuang et al. [30]. The results were also similar to those reported by various other authors [17,31,32]. All cricket chitosan showed slightly sharper peaks, indicating that cricket chitosan may be slightly more crystalline than commercial shrimp chitosan.

Figure 3c displays the TGA thermograms of cricket chitosan. All cricket chitosan and CSC showed similar patterns of weight loss. A first weight loss was seen at temperatures ranging from 50 to 100 °C, which corresponds to a loss of moisture of around 5% [33]. All cricket chitosan and CSC started to decompose (at 280 °C) and lost weight rapidly until 400 °C. This degradation is due to the amine and acetyl groups of chitosan depolymerizing and breaking down [17].

Figure 3d shows the DSC thermograms of the various cricket chitosan. The DSC thermogram of CSC exhibited an endothermic peak in the temperature range of 50–120 °C. This peak can be attributed to the evaporation of water or moisture contained in chitosan [34]. On the other hand, this endothermic peak may overlap with the one corresponding to the glass transition temperature (Tg) of chitosan. Dong et al. (2004) reported the Tg of chitosan to be around 140–150 °C [35], which aligns well with the findings of Ogura et al. (1980), who reported a Tg of approximately 150 °C [36]. Additionally, Kim et al. (2002) stated that the Tg of chitosan occurs within a broader range of 100–200 °C [37]. However, in certain studies, the Tg of chitosan was reported to be higher, reaching up to 203 °C [38]. The variation in the Tg of chitosan reported in different studies can be explained by the plasticizing effect of water and the high hygroscopicity of chitosan, which hinder the accurate determination of the glass transition temperature and cause significant dispersion in the experimental data [35,39]. In addition, this endothermic peak is followed by an exothermic peak at 270 °C, which refers to the thermal decomposition of chitosan [17], and corresponds to the TGA measurements (280 °C) in the present work. Importantly, all cricket chitosan exhibited essentially the same pattern when compared to CSC.

Figure 3e compares the ^1^H-NMR spectra of CSC with three cricket-extracted chitosan (GBC, TMC, and ADC). Each chitosan sample (30 mg) was dissolved in 1% acetic acid in deuterium oxide (D_2_O). The cricket-derived chitosan samples exhibited three distinct proton signal regions: δ 2.97 (methine proton, H-2), δ 3.51 (methine proton, H-5), and δ 3.70 (methylene protons, H-6 and methine protons, H-3,4). These proton signal regions are consistent with previously published chitosan ^1^H-NMR spectra [40,41]. In contrast, the CSC spectrum displayed signals at a lower field: δ 3.05 (methine proton, H-2), δ 3.61 (methine proton, H-5), and δ 3.79 (methylene protons, H-6 and methine protons, H-3,4). The downfield shift in the ^1^H-NMR signals in CSC is attributed to its higher molecular weight, which significantly distinguishes it from the cricket-derived chitosan [42].

The characterization results discussed above revealed notable similarities between various cricket chitosan and CSC, suggesting potential applications for cricket chitosan. However, to gain a more comprehensive understanding, further characterization, including additional analyses using gel permeation chromatography and other microanalytical techniques, is recommended.

**Figure 4:**
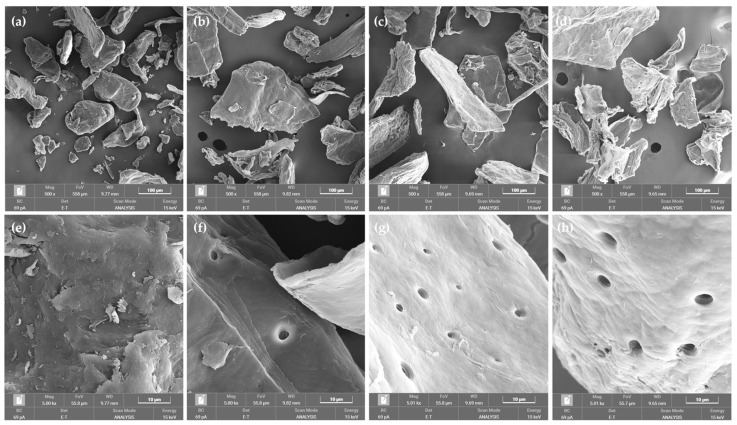
SEM images of chitosan pellets observed at 500× magnification, including commercial shrimp chitosan (CSC) (**a**), *G. bimaculatus* chitosan (GBC) (**b**), *T. mitratus* chitosan (TMC) (**c**), and *A. domesticus* chitosan (ADC) (**d**), and at 5000× magnification for CSC (**e**), GBC (**f**), TMC (**g**), and ADC (**h**).

SEM images of cricket chitosan are shown in Figure 4. CSC and cricket chitosan have various sizes, irregular shapes, and smooth surfaces, with no sign of mineral impurities. In terms of dimensions, *G. bimaculatus* chitosan (GBC) and *T. mitratus* chitosan (TMC) particles were larger and flatter than CSC and *A. domesticus* chitosan (ADC), as shown in Figure 4a–d. The main difference between CSC and cricket chitosan however is the number of pores with a diameter of less than 10 μM that cricket chitosan contain, as shown in Figure 4e–h. This observation has the same results as the previous study by Psarianos et al., which showed that, especially chitosan from the house cricket, had a smooth surface with many pores of less than 10 μM in diameter, which were not found in commercial chitosan [33]. These pores are believed to stem from spiracles in the tracheae that open out on the cuticle laterally along the insect’s body [43].

### 3.3. Solubility of Cricket Chitosan

**Figure 5:**
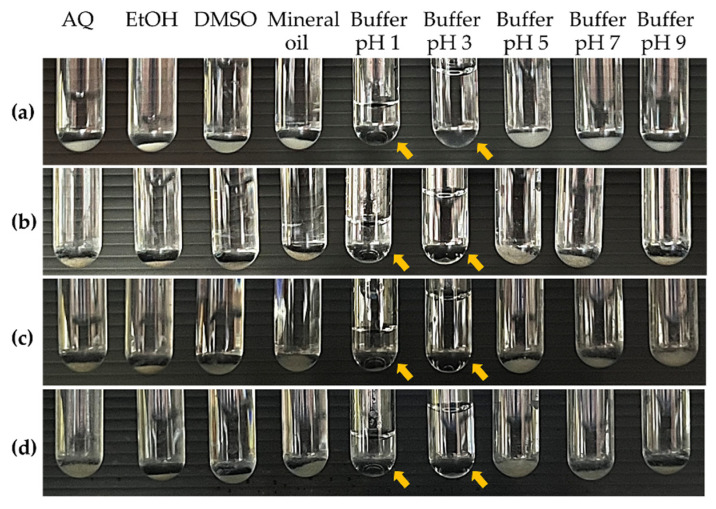
Solubility of commercial shrimp chitosan (CSC) (**a**) and chitosan extracted from *G. bimaculatus* (GBC) (**b**), *T. mitratus* (TMC) (**c**), and *A. domesticus* (ADC) (**d**) in various solvents, including deionized water (AQ), absolute ethanol (EtOH), dimethyl sulfoxide (DMSO), mineral oil, and buffer solutions with pHs ranging from 1 to 9. Yellow arrows pointing to transparent solutions indicate solubility of chitosan samples in respective solvents.

The solubility of chitosan in water is a constraint when considering its use in cosmetic formulations. The solubility of cricket chitosan compared to CSC in different solvents is illustrated in Figure 5 and shown in Table 2. All cricket chitosan are insoluble in deionized water, absolute ethanol, DMSO, and mineral oil, including in buffers with a pH higher than 5, but are soluble in buffer pH 1 and in buffer pH 3. These findings confirm that chitosan is insoluble in organic solvents [44] and water, but soluble in acidic solvents such as diluted hydrochloric and acetic acids. When chitosan is in an acidic environment, the amino groups become protonated, giving the molecule a positive charge [45]. Moreover, previous studies also show that chitosan was insoluble and precipitated at a pH over 5 [46]. Typically, chitosan can be dissolved in mild organic acids, indicating a deacetylation level of approximately 40–45% [3]. The solubility of ADC, which also had the lowest molecular weight (see below), was the highest compared to GBC and TMC (14.51 ± 1.68 and 3.77 ± 0.05 mg/mL in buffer pH 1 and buffer pH 3, respectively), confirming that the molecular weight of chitosan significantly affects its physicochemical characteristics, including solubility and hydrophilicity [3,47].

### 3.4. Water and Fat Binding Capacities

The water and fat binding capacities of the cricket chitosan were determined next and were around 554–620% and 526–694%, respectively, as presented in Table 2. ADC shows both the highest water and fat binding capacity compared to the other chitosan. This result is similar to a previous study showing that chitosan from prawn shells showed water and fat binding capacities of 627% and 574%, respectively [18]. In general, the lower the molecular weight of the chitosan, the lower the crystallinity, and the greater the increase in water binding capacity [18]. When focusing on the molecular weight and water binding capacity of ADC versus CSC, the results confirm this hypothesis. However, the water binding capacity of GBC and TMC does not relate to molecular weight when compared with CSC. These results may be caused by the particle size and shape of chitosan. ADC and CSC particle have smaller sizes and more irregular shapes than GBC and TMC, as shown in Figure 4a–d. The higher water binding capacity of CSC and ADC could be attributed to a large surface area to absorb water [48].

### 3.5. Molecular Weight

The molecular weight of cricket chitosan was found to be 919.35–1000 kDa, as shown in Table 2, which is similar to a previous study [19,49]. When compared with CSC however the molecular weight of cricket chitosan was lower than those of CSC. This suggests that the chitosan chains of all cricket chitosan are shorter than that of CSC. A previous study reported that the molecular weight of chitosan is significantly influenced by both its source and the specific extraction and production methods [50]. Notably, acid and alkali treatments were shown to induce deacetylation and molecular weight reduction [51]. Therefore, a likely explanation for the lower molecular weight of cricket chitosan in the present study is the harsh extraction process, which involved the use of a strong base (NaOH) for the deproteinization and deacetylation processes, along with a strong acid (HCl) for the demineralization process. This led to chitosan degradation, ultimately resulting in lower molecular weights.

### 3.6. Irritation Potency and Cytotoxicity of Cricket Chitosan

**Figure 6:**
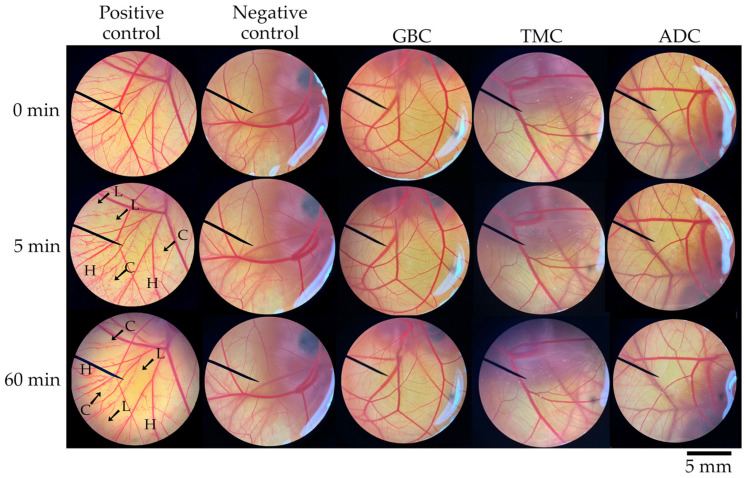
Photographs of chorionallantoic membrane (CAM) before (0 min) and after exposure to positive control (1% *w*/*v* sodium lauryl sulfate aqueous solution), negative control (normal saline solution), and chitosan extracted from *G. bimaculatus* (GBC), *T. mitratus* (TMC), and *A. domesticus* (ADC) at 5 min and 60 min. Letter ‘H’ denotes vascular hemorrhage, ‘L’ denotes vascular lysis, and ‘C’ denotes vascular coagulation.

Next, we evaluated the irritation potency of chitosan extracted from crickets to confirm that the extraction process produced non-irritant materials. The extraction process of chitosan involves treating the material with a strong acid and alkali solution, specifically HCl to remove minerals and NaOH to remove proteins. Figure 6 illustrates the CAM exposures to each cricket chitosan, and Table 3 comprises the irritation scores. The results indicate that all cricket chitosan can be considered as safe and did not induce any signs of irritation.

**Figure 7:**
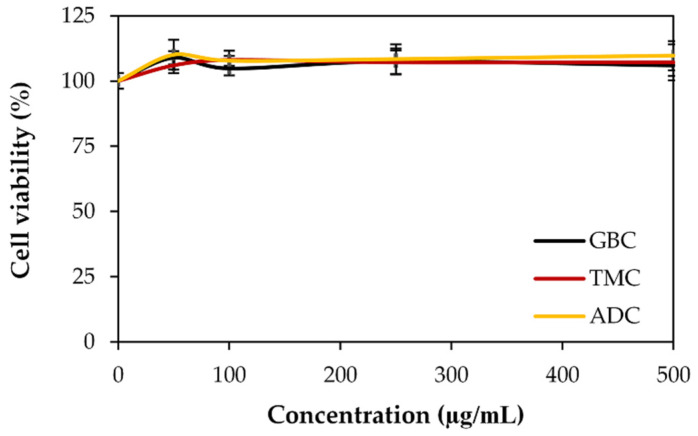
Cytotoxicity of cricket chitosan on human dermal fibroblasts. Percentages of HNDF cell viability after 24 h treated with different concentrations of chitosan extracted from *G. bimaculatus* (GBC), *T. mitratus* (TMC), and *A. domesticus* (ADC). Results presented as mean ± standard deviation (*n* = 4).

The MTT assay was used as a quantitative method that measures mitochondrial activity in living cells to verify the safety of cricket chitosan for dermal products. Figure 7 shows that when exposed to all cricket chitosan solutions, NHDF cell viability was over 100%, indicating that there were no cytotoxic effects. Interestingly, all cricket chitosan showed a significantly higher cell viability than the control (*p*-value less than 0.05). This result indicates that cricket chitosan can enhance NHDF proliferation. The findings were in line with previous studies showing that chitosan can stimulate cell proliferation. Chitosan with a high level of deacetylation greatly enhanced fibroblast proliferation [52] and increased the early stages of cell proliferation and facilitated fibroblast migration, which included collagen synthesis [53]. In actuality, differentiation and proliferation of keratinocytes and fibroblasts can stimulate the production of collagen, lipids, and other extracellular matrix constituents in the skin [54].

**Figure 8:**
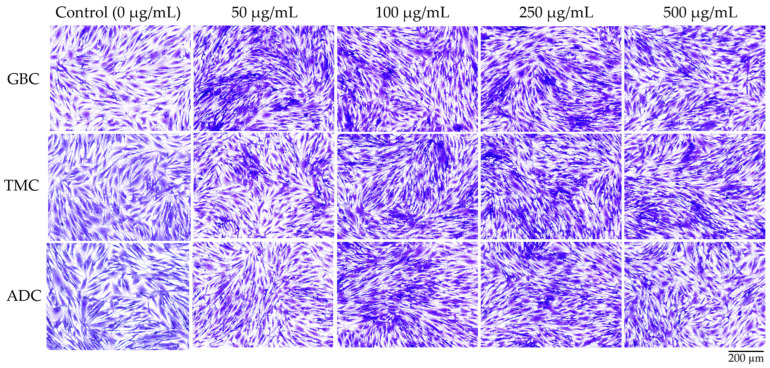
Cell morphology and density of human dermal fibroblast (NHDF) cells after 24 h exposure to chitosan solutions derived from *G. bimaculatus* (GBC), *T. mitratus* (TMC), and *A. domesticus* (ADC). Cells stained with crystal violet and visualized at 200× magnification, with scale bar of 50 μm. Chitosan concentrations ranged from 50 to 500 μg/mL. Control group exposed to phosphate buffer pH 7.4.

The safety of cricket chitosan was further verified by evaluating the morphology of NHDF cells using crystal violet staining, as illustrated in Figure 8. The NHDF cells that were exposed to the chitosan solution did not show any differences in terms of morphology when compared to the control cells. Simultaneously, NHDF cells treated with cricket chitosan had a higher density than the control cells. The results of the present study indicate that cricket chitosan exhibited excellent biocompatibility and can be considered safe for dermal application.

### 3.7. Development of CNPs

**Figure 9:**
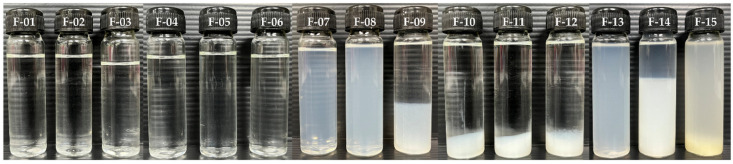
External appearance of CNPs from commercial shrimp chitosan (CSC) at concentration of 0.1% *w*/*w* along with various crosslinking agents, including citric acid (F-01: 0.075% *w*/*w*; F-02: 0.25% *w*/*w*; and F-03: 0.75% *w*/*w*), gum arabic (F-04: 0.075% *w*/*w*; F-05: 0.25% *w*/*w*; and F-06: 0.75% *w*/*w*), sodium citrate (F-07: 0.075% *w*/*w*; F-08: 0.25% *w*/*w*; and F-09: 0.75% *w*/*w*), and sodium tripolyphosphate (F-10: 0.075% *w*/*w*; F-11: 0.25% *w*/*w*; and F-12: 0.75% *w*/*w*), and the CNPs from various concentrations of CSC, e.g.,(F-08: 0.10% *w*/*w*; F-13: 0.25% *w*/*w*; F-14: 0.75% *w*/*w*; and F-15: 1.00% *w*/*w*) with 0.25% *w*/*w* of sodium citrate as a crosslinking agent.

CNPs, prepared by an ionotropic gelation method, were optimized by the interaction between positive-charged chitosan and various negatively charged crosslinking agents. Figure 9 shows the external appearance of the CNP formulation from CSC crosslinked with citric acid, gum arabic, sodium citrate, and sodium tripolyphosphate. Formulations F-01 to F-06 (see figure caption in Figure 9) show a clear solution and formulations F-07 and F-08 give a translucent solution, whereas formulations F-08 to F-12 exhibited sedimentation of the suspension after 24 h of preparation. The particle size of CSC-citric acid and CSC-gum arabic showed a dose dependency on particle size, as shown in Table 4. The increase in citric acid and gum arabic concentration causes a decrease in pH, particle size, and zeta potential. Previous studies have shown that as acetic acid levels increase, the particle size of GB-CNP decreases [55]. This phenomenon may be explained by the effect of pH on positive charge density and the solubility of the chitosan polymer [56]. In essence, chitosan is susceptible to interacting with negatively charged polyions due to its behavior as a polycation at low pH values [57]. When the pH decreased due to chitosan–citric acid crosslinking, the CNP size decreased [58]. In contrast, CSC-sodium citrate at a concentration of 0.75% *w*/*w* sodium citrate (formulation F-09) agglomerated and sedimented (pH = 5.93). Similarly, all formulations from CSC-sodium tripolyphosphate (formulation F-10, F-11, and F-12) contained sediment due to their pH values of 5.0, 5.2, and 7.3, respectively. pH and ionic strength have an impact on the size and polydispersity of CNP formation [59,60]. According to reports, chitosan–sodium tripolyphosphate and chitosan–citrate solutions were turbid at pH 4.3 and precipitated in the pH region above 4.3 [56]. The minimum size (209.5 ± 0.7 to 256.4 ± 2.20 nm) and narrowed PDI (0.21± 0.00 to 0.28 ± 0.02) of the CNPs were obtained from formulations F-07 and F-08 for a CSC concentration of 0.1% (*w*/*w*) and sodium citrate concentrations of 0.075% (*w*/*w*) and 0.25% (*w*/*w*), respectively.

The effect of CSC concentration on CNP size was also optimized. F-14 and F-15 had sediments at CSC concentrations higher than 0.75% (*w*/*w*). Formulation F-08 (CSC 0.10% *w*/*w*) and F-13 (CSC 0.25% *w*/*w*) were translucent with a particle size of 209.5 ± 0.7 to 494.2 ± 4.7 nm, respectively, as shown in Table 4. Therefore, the optimum concentration of CSC in CNP production falls within the range of 0.10 to 0.25% *w*/*w*. This result is related to a previous study where the optimum concentration of chitosan that produced nanoparticles by the ionotropic gelation method was found to be 0.05–0.15% *w*/*w* [24,58,61].

**Figure 10:**
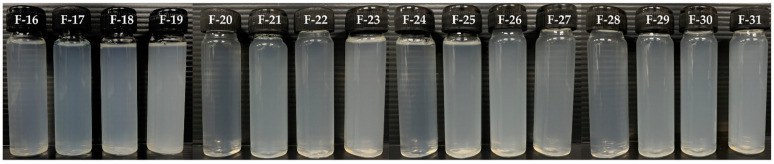
External appearance of CNPs from various types of chitosan, including commercial shrimp chitosan (F-16: 0.10% *w*/*w*; F-17: 0.15% *w*/*w*; F-18: 0.20% *w*/*w*; and F-19: 0.25% *w*/*w*), *G. bimaculatus* chitosan (F-20: 0.10% *w*/*w*; F-21: 0.15% *w*/*w*; F-22: 0.20% *w*/*w*; and F-23: 0.25% *w*/*w*), *T. mitratus* chitosan (F-24: 0.10% *w*/*w*; F-25: 0.15% *w*/*w*; F-26: 0.20% *w*/*w*; and F-27: 0.25% *w*/*w*), and *A. domesticus* chitosan (F-28: 0.10% *w*/*w*; F-29: 0.15% *w*/*w*; F-30: 0.20% *w*/*w*; and F-31: 0.25% *w*/*w*).

CNPs from cricket chitosan (formulations F-20 to F-31) and from CSC (formulations F-16 to F-19) were successfully prepared at various concentrations, as shown in Table 5. Figure 10 illustrates that the physical appearance of all CNP formulations was translucent and had no precipitation after preparation (0 days) and after stability testing (60 days). Table 5 shows the particle size, polydispersity index, and zeta potential of the CNPs from the cricket chitosan formulation. After preparation, all formulations exhibited particle sizes in the nanometer range (369.1 ± 3.2 to 686.0 ± 5.1 nm) with a PDI less than 0.5 and a strong positive charge (24.8 ± 2.0 to 34.7 ± 4.3 mV). As the concentration of cricket chitosan increased, the size of the CNPs and surface charge also increased. This agrees with previous reports, which found that with an increasing concentration of chitosan, particle size and surface charge also increased [62]. At 0.25% *w*/*w* of chitosan, CNPs from ADC (formulation F-31) were the smallest (522.0 ± 12.1 nm) compared to those from CSC (494.2 ± 4.7 nm), GBC (666.4 ± 8.9 nm), and TMC (686.0 ± 5.1 nm) from formulations F-19, F-23, and F-27, respectively, (*p* < 0.05). After 60 days of stability testing, the sizes of the CNPs from the cricket chitosan decreased, whereas PDI and surface charge remained constant, as shown in Table 5. This result is similar to a previous report by Morris et al. [63]. In contrast, a decrease in the sizes of the CNPs was seen after 1 month of storage. The observed phenomenon can be explained by the decrease in the molar mass of the chitosan, possibly caused by hydrolysis leading to the breaking of polymer chains. Since the zeta potential determines the stability of the nanoparticles in the fluid [12], the CNPs from cricket chitosan, which had a larger zeta potential, demonstrated higher stability in aqueous media than the CNPs from CSC.

## 4. Conclusions

Chitosan from three edible cricket species commonly farmed in Thailand was successfully extracted from defatted cricket powder and isolated through demineralization, deproteinization, and deacetylation processing steps, with a yield of 4.35 to 5.22%. FT-IR, TGA, DSC, and ^1^H-NMR results revealed similarities between cricket chitosan and CSC. The XRD diffractograms showed that all cricket chitosan materials had a slightly higher crystallinity than CSC. The morphology of the cricket chitosan revealed numerous pores, which were absent in CSC. All chitosan were soluble in acidic buffers. The molecular weight of the cricket chitosan was revealed to be 919.35–1000 kDa, lower than CSC. Cricket chitosan exhibited slight differences in both water and fat binding capacities, while ADC demonstrated the highest capacities for both water and fat binding compared to CSC. The ionotropic gelation method successfully produced CNPs from the cricket chitosan. Cricket chitosan nanoparticles were noticeably larger than those from CSC. However, compared to other cricket chitosan, the ADC was found to be the most suitable for CNP development due to its smallest particle size, narrow polydispersity index, and acceptable positive surface charge. The results of this study suggest that cricket chitosan could be used as a nano delivery system for dermal dosage forms. Further exploration of CNPs from cricket chitosan development by incorporating active ingredients into the nanoparticle is recommended to characterize its entrapment efficiency, release profile, and skin penetration.

## Figures and Tables

**Table 1:** Yields of deproteinized chitin, demineralized chitin, and deacetylated chitosan from *G. bimaculatus*, *T. mitratus*, and *A. domesticus*.

Cricket Species	Production Yield (Mean ± SD)		
	Deproteinized Chitin	Demineralized Chitin	Deacetylated Chitosan
*G. bimaculatus*	8.71 ± 1.31 ^ab,B^	7.43 ± 1.17 ^ns,AB^	5.22 ± 0.99 ^ns,A^
*T. mitratus*	7.77 ± 1.12 ^a,B^	6.65 ± 0.97 ^ns,B^	4.35 ± 0.72 ^ns,A^
*A. domesticus*	9.91 ± 0.09 ^b,C^	7.50 ± 1.09 ^ns,B^	5.17 ± 0.89 ^ns,A^

Note: Results expressed as mean ± standard deviation (*n* = 3) and evaluated by one-way ANOVA and Tukey’s test (*p* < 0.05). Different small letters in each column and capital letters in each row indicate significant difference between means, while ‘ns’ represents no significant difference.

**Table 2:** Solubility, molecular weight, and binding capacity of chitosan.

Samples	Solubility (mg/mL)		Molecular Weight (kDa)	Binding Capacity (%*w*/*w*)	
	Buffer pH 1	Buffer pH 3		Water Binding	Fat Binding
Commercial shrimp chitosan	9.95 ± 0.67 ^a^	2.39 ± 0.12 ^a^	1428.50 ± 158.35 ^b^	620.67 ± 6.11 ^c^	550.67 ± 17.01 ^a^
*G. bimaculatus chitosan*	10.15 ± 0.47 ^a^	2.38 ± 0.13 ^a^	1000.47 ± 47.18 ^a^	596.00 ± 8.72 ^b^	528.00 ± 20.30 ^a^
*T. mitratus chitosan*	13.68 ± 1.32 ^b^	3.08 ± 0.23 ^b^	933.01 ± 9.37 ^a^	554.00 ± 11.14 ^a^	526.67 ± 5.03 ^a^
*A. domesticus chitosan*	14.51 ± 1.68 ^b^	3.77 ± 0.05 ^c^	919.35 ± 26.15 ^a^	756.00 ± 3.46 ^d^	694.67 ± 10.07 ^b^

Note: Results expressed as mean ± standard deviation (*n* = 3). Different letters (a–d) indicate significant differences among various chitosan, analyzed using one-way ANOVA followed by Tukey’s multiple comparison test (*p* < 0.05).

**Table 3:** Irritation score and irritation potency of chitosan.

Samples	Irritation Score	Irritation Potency
Positive control	19.2 ± 0.1 ^a^	Severe irritation
Negative control	0.0 ± 0.0 ^b^	No irritation
*G. bimaculatus* chitosan	0.0 ± 0.0 ^b^	No irritation
*T. mitratus* chitosan	0.0 ± 0.0 ^b^	No irritation
*A. domesticus* chitosan	0.0 ± 0.0 ^b^	No irritation

Note: Results expressed as mean ± standard deviation (*n* = 2). Different letters (a and b) indicate significant differences among irritation scores of each sample, analyzed using one-way ANOVA followed by Tukey’s multiple comparison test (*p* < 0.05).

**Table 4:** Final concentrations of commercial shrimp chitosan and crosslinking agents, pH, particle size, polydispersity index, and zeta potential of chitosan nanoparticle formulations.

Formulation	Commercial Shrimp Chitosan (CSC): Type and Amount of Crosslinking Agent (% *w*/*w*)	pH	Particle Size (nm)	Polydispersity Index (PDI)	Zeta Potential (mV)
F-01	CSC 0.10:CA 0.075	3.34	581.4 ± 12.5 ^b,c^	0.89 ± 0.07 ^c^	32.7 ± 1.8 ^c^
F-02	CSC 0.10:CA 0.250	2.86	300.1 ± 19.8 ^a^	0.86 ± 0.09 ^c^	21.8 ± 2.1 ^b^
F-03	CSC 0.10:CA 0.750	2.48	243.3 ± 18.7 ^a^	0.91 ± 0.07 ^c^	13.5 ± 0.9 ^a,b^
F-04	CSC 0.10:GA 0.075	3.86	860.6 ± 55.7 ^d^	1.00 ± 0.00 ^c^	57.7 ± 1.1 ^d^
F-05	CSC 0.10:GA 0.250	3.86	661.0 ± 59.1 ^c^	1.00 ± 0.00 ^c^	55.1 ± 4.0 ^d^
F-06	CSC 0.10:GA 0.750	3.82	617.7 ± 24.4 ^c^	0.95 ± 0.06 ^c^	40.7 ± 6.1 ^c^
F-07	CSC 0.10:SC 0.075	4.52	256.4 ± 2.20 ^a^	0.28 ± 0.02 ^a^	21.4 ± 0.7 ^b^
F-08	CSC 0.10:SC 0.250	4.96	209.5 ± 0.7 ^a^	0.21 ± 0.00 ^a^	12.2 ± 1.2 ^a^
F-13	CSC 0.25:SC 0.250	5.00	494.2 ± 4.7 ^b^	0.58 ± 0.01 ^b^	16.6 ± 0.5 ^a,b^

Note: Results expressed as mean ± standard deviation (*n* = 3). Different letters (a–d) indicate significant differences among various formulations, analyzed statistically using one-way ANOVA followed by Tukey’s multiple comparison test (*p* < 0.05).

**Table 5:** Final concentrations of cricket chitosan and sodium citrate, particle size, polydispersity index, and zeta potential of chitosan nanoparticle formulations.

Formulation	Type and Amount of Chitosan: Sodium Citrate (% *w*/*w*)	Particle Size (nm)		Polydispersity Index (PDI)		Zeta Potential (mV)	
		Day 0	Day 60	Day 0	Day 60	Day 0	Day 60
F-16	CSC 0.10:SC 0.25	216.0 ± 4.7 ^a^	255.5 ± 3.1 ^a,^*	0.185 ± 0.014 ^a^	0.248 ± 0.005 ^a,^*	9.7 ± 0.4 ^a^	16.6 ± 1.7 ^a,^*
F-17	CSC 0.15:SC 0.25	273.1 ± 3.2 ^b^	282.1 ± 6.7 ^b^	0.271 ± 0.004 ^b^	0.291 ± 0.010 ^a^	13.0 ± 1.3 ^b^	20.6 ± 1.5 ^b,^*
F-18	CSC 0.20:SC 0.25	380.3 ± 4.5 ^c^	379.4 ± 6.8 ^c^	0.481 ± 0.018 ^c^	0.369 ± 0.021 ^b,^*	13.7 ± 1.1 ^b^	21.8 ± 0.8 ^b,^*
F-19	CSC 0.25:SC 0.25	494.2 ± 4.7 ^d^	532.8 ± 0.6 ^d,^*	0.576 ± 0.009 ^d^	0.353 ± 0.026 ^b,^*	16.6 ±0.5 ^c^	27.9 ± 1.3 ^c,^*
F-20	GBC 0.10:SC 0.25	369.1 ± 3.2 ^a^	296.0 ± 5.7 ^a,^*	0.255 ± 0.009 ^a^	0.219 ± 0.017 ^a^	29.9 ± 3.5 ^a^	28.0 ± 1.6 ^a^
F-21	GBC 0.15:SC 0.25	547.1 ± 3.8 ^b^	463.0 ± 7.1 ^b,^*	0.477 ± 0.005 ^b^	0.402 ± 0.015 ^b,^*	30.0 ± 2.4 ^a^	30.8 ± 1.8 ^a^
F-22	GBC 0.20:SC 0.25	641.6 ± 18.1 ^c^	464.0 ± 4.5 ^b,^*	0.437 ± 0.043 ^b^	0.428 ± 0.001 ^b^	33.8 ± 3.6 ^a^	28.8 ± 3.1 ^a^
F-23	GBC 0.25:SC 0.25	666.4 ± 8.9 ^c^	479.2 ± 9.7 ^b,^*	0.455 ± 0.021 ^b^	0.410 ± 0.028 ^b^	34.7 ± 4.3 ^a^	29.2 ± 0.8 ^a^
F-24	TMC 0.10:SC 0.25	425.1 ± 2.8 ^a^	339.2 ± 3.0 ^a,^*	0.225 ± 0.017 ^a^	0.221 ± 0.008 ^a^	28.0 ± 1.6 ^a^	30.4 ± 1.3 ^a^
F-25	TMC 0.15:SC 0.25	509.0 ± 4.1 ^b^	404.1 ± 1.2 ^b,^*	0.458 ± 0.014 ^b^	0.273 ± 0.016 ^ab,^*	30.6 ± 1.8 ^a^	30.5 ± 1.0 ^a^
F-26	TMC 0.20:SC 0.25	635.5 ± 12.9 ^c^	426.2 ± 16.5 ^b,^*	0.411 ± 0.045 ^b^	0.314 ± 0.015 ^bc^	32.2 ± 2.7 ^a^	31.9 ± 2.5 ^a^
F-27	TMC 0.25:SC 0.25	686.0 ± 5.1 ^d^	552.6 ± 24.4 ^c,^*	0.482 ± 0.009 ^b^	0.348 ± 0.041 ^c^	32.3 ± 2.1 ^a^	36.8 ± 4.6 ^a^
F-28	ADC 0.10:SC 0.25	376.1 ± 3.0 ^a^	305.7 ± 3.4 ^a,^*	0.220 ± 0.021 ^a^	0.213 ± 0.026 ^a^	24.8 ± 2.0 ^a^	26.8 ± 1.9 ^a,^*
F-29	ADC 0.15:SC 0.25	398.4 ± 3.8 ^b^	378.2 ± 15.3 ^b^	0.382 ± 0.008 ^b^	0.305 ± 0.022 ^b,^*	25.3 ± 2.1 ^ab^	29.4 ± 3.4 ^a,^*
F-30	ADC 0.20:SC 0.25	426.7 ± 6.1 ^c^	382.5 ± 2.8 ^b,^*	0.340 ± 0.060 ^b^	0.333 ± 0.041 ^b^	30.2 ± 3.3 ^ab^	29.3 ± 1.0 ^a^
F-31	ADC 0.25:SC 0.25	522.0 ± 12.1 ^d^	392.8 ± 7.0 ^b,^*	0.388 ± 0.026 ^b^	0.368 ± 0.003 ^b^	34.2 ± 4.4 ^b^	30.3 ± 2.1 ^a^

Results expressed as mean ± standard deviation (*n* = 3). Different letters (a, b, c, and d) indicate significant differences among mean of each sample within type of chitosan, analyzed using one-way ANOVA followed by Tukey’s multiple comparison test (*p* < 0.05). Asterisk * indicates statistical significance of differences among formulations at day 0 and day 60, analyzed using paired *t*-test comparisons (*p* < 0.05).

## Data Availability

Data available upon request.
